# Comparative Analysis of Gene Expression Profiles in the Adipose Tissue of Obese Adult Mice With Rapid Infantile Growth After Undernourishment *In Utero*

**DOI:** 10.3389/fendo.2022.818064

**Published:** 2022-02-24

**Authors:** Misako Suzuki, Yukiko Kohmura-Kobayashi, Megumi Ueda, Naomi Furuta-Isomura, Masako Matsumoto, Tomoaki Oda, Kenta Kawai, Toshiya Itoh, Madoka Matsuya, Megumi Narumi, Naoaki Tamura, Toshiyuki Uchida, Kazuki Mochizuki, Hiroaki Itoh

**Affiliations:** ^1^ Department of Obstetrics and Gynecology, Hamamatsu University School of Medicine, Hamamatsu, Japan; ^2^ Laboratory of Food and Nutritional Sciences, Department of Local Produce and Food Sciences, Faculty of Life and Environmental Sciences, University of Yamanashi, Yamanashi, Japan

**Keywords:** obesity, pregnancy, adipose tissue, inflammation, catch-up growth, Developmental Origins of Health and Disease (DOHaD), low birth weight, macrophage

## Abstract

Rapid infantile growth (RG) markedly increases the risk of obesity and metabolic disorders in adulthood, particularly among neonates born small. To elucidate the molecular mechanisms by which RG following undernourishment *in utero* (UN) contributes to the deterioration of adult fat deposition, we developed a UN mouse model using maternal energy restriction, followed by RG achieved by adjustments to 4 pups per litter soon after birth. A high-fat diet (HFD) was fed to weaned pups treated or not (Veh) with tauroursodeoxycholic acid (TU). UN-RG pups showed the deterioration of diet-induced obesity and fat deposition, which was ameliorated by TU. We performed a microarray analysis of epididymal adipose tissue and two gene enrichment analyses (NN-Veh *vs* UN-RD-Veh and UN-RG-Veh *vs* UN-RG-TU). The results obtained identified 4 common gene ontologies (GO) terms of inflammatory pathways. In addition to the inflammatory characteristics of 4 GO terms, the results of heatmap and principal component analyses of the representative genes from 4 GO terms, genes of interest (GOI; *Saa3, Ubd, S100a8, Hpx, Casp1, Agt, Ptgs2*) selected from the 4 GO terms, and immunohistochemistry of macrophages collectively suggested the critical involvement of inflammation in the regulation of fat deposition in the responses to UN and TU. Therefore, the present results support the ‘Developmental Origins of Metaflammation’, the last word of which was recently proposed by the concept of metabolic disorders induced by low-grade systemic inflammation.

## Introduction

We are in the midst of a worldwide obesity epidemic. The global prevalence of obesity has doubled since 1980 (1) and is now an international public health issue (2). Obesity is frequently associated with impaired glucose tolerance, dyslipidemia, and hypertension, and the combination of these metabolic disorders is associated with atherosclerosis-related diseases. Therefore, the concept of metabolic syndrome has been proposed (3). Increasing evidence has demonstrated that environmental disruptions in the early susceptible period affect health and promote a predisposition to non-communicable diseases (NCDs) in later life, based upon which the concept of the Developmental Origins of Health and Disease (DOHaD) was established (4, 5). Eriksson et al. (2001) identified a high body mass index (BMI) at 7 years old as a risk factor for adult obesity with low-birth weights in 3,659 individuals born at Helsinki University Hospital between 1924 and 1933 (6). Ong et al. conducted systematic reviews and demonstrated that rapid infantile weight gain was consistently associated with an increased risk of obesity later in life (7). Ekelund et al. (2007) showed that rapid infantile weight gain was associated with clustered metabolic disruptions in 128 affected individuals at the age of 17 years (8). Martin et al. (2017) performed a systemic review and found that longer-term health outcomes found catch-up growth were associated with a higher body mass, BMI, or cholesterol in later life; however, catch-up growth following low birth weight may have short-term benefits for individuals with low birth weight (9). Singhal et al. (2017) reported that growth acceleration in healthy infants born at term (either normal or small for gestation) may be associated with an increased long-term risk of obesity and NCDs; however, the benefits of rapid infant weight gain for later neurodevelopment supports its encouragement in infants born preterm (10). These findings led us to the concept that rapid infantile growth (RG) in term newborns, particularly with low-birth weights, is causatively associated with a predisposition to obesity and metabolic syndrome in later life. However, to the best of our knowledge, the underlying molecular mechanisms by which RG in small newborns programs a predisposition to the deterioration of fat deposition have not yet been elucidated. Indeed, risk of NCDs resulted from RG in small newborns applies well to ‘Mismatch hypothesis’ (11) (12), one of central hypotheses of DOHaD theory. In the present study, we hypothesized the presence of specific pattens in gene expression profiles of the adipose tissue of pups with RG and undernutrition *in utero* (UN).

We established a mouse model of UN using maternal energy restriction, which develops the phenotypes of various NCDs (13–15), and subsequently demonstrated that diet-induced fat deposition in adipose tissue and the liver was significantly greater in UN pups than in normal nutrition (NN) pups (13, 16–18). Oral treatments with the hydrophilic secondary bile acid tauroursodeoxycholic acid (TUDCA), an endoplasmic reticulum (ER) stress alleviator, markedly ameliorated developmentally-induced hepatic steatosis in UN pups, but not in NN pups (17, 18). Therefore, modifications to this animal model of UN are promising for investigating the effects of RG on developmentally-programed fat deposition and its amelioration by TUDCA (TU).

The overall aim of the present study was to examine the characteristics of gene expression profiles associated with the changes in the amount of adult adipose tissue deposition, i.e. deteriorations induced by RG after UN and improvements by TU. The specific objectives of the present study were 1) to develop a mouse model of RG soon after birth by reducing the number of pups per litter to 4 pups (UN-RG) compared to 8 pups per litter for NN, 2) to investigate the effects of TU on UN-RG pups and NN pups fed a high-fat diet (HFD), 3) to perform a microarray analysis followed by a gene enrichment analysis of epididymal fat tissues (NN-Veh *vs* UN-RG-Veh and UN-RG-Veh *vs* UN-RG-TU) in order to identify common gene ontologies (GO) terms, 4) to conduct heatmap and principal component analyses of the common GO identified, 5) to identify genes of interest (GOI) from these GO terms and subject them to quantitative RT-PCR, and 6) to immunohistochemically assess the infiltration of macrophages in epididymal adipose tissue in consideration of the characteristics of the GO terms and GOI identified.

## Materials and Methods

### Mouse Model of UN-RG

**Figure 1:**
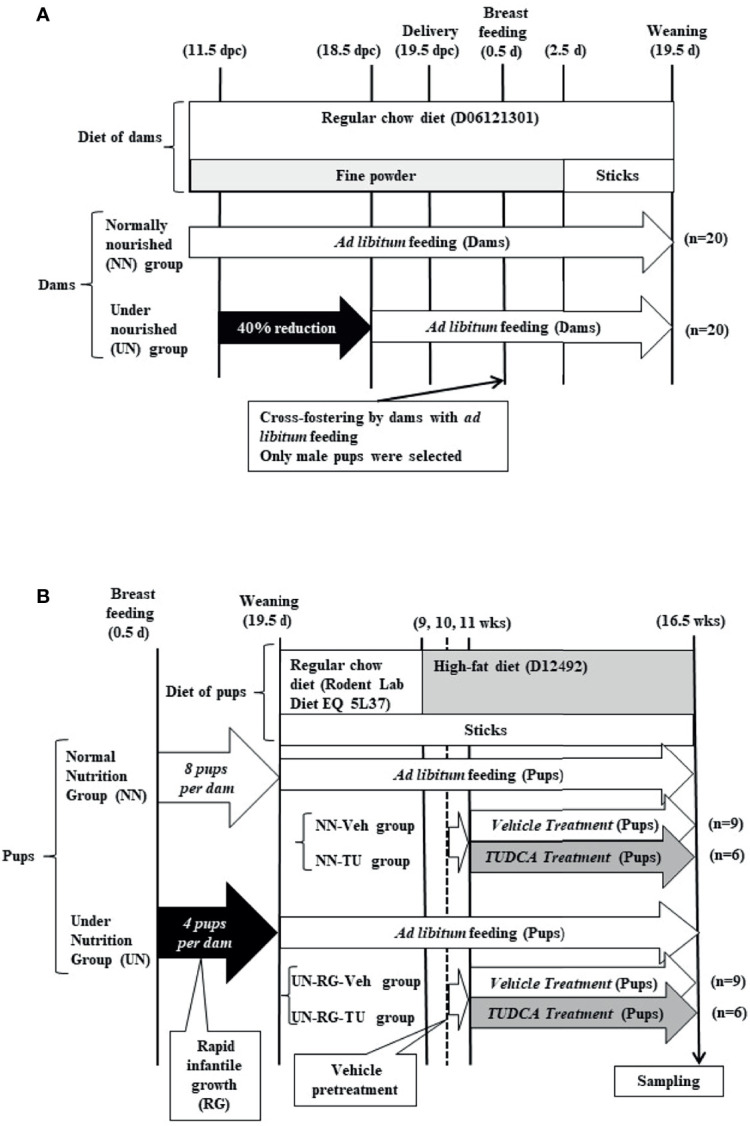
Schematic illustration of experimental procedures in dams **(A)** and pups **(B)**.

The procedures used are shown in **Figures 1A, B**. We modified our previously reported animal model of maternal energy restriction (13, 14, 16–19) such that pups were subjected to RG during the lactation period until weaning (19.5 d) following UN by maternal energy restriction. In brief, pregnant C57Bl/6 NCrSlc mice were purchased at 7.5 days post coitum (dpc) from Japan SLC, Inc. (Hamamatsu, Japan) and housed individually with free access to water under a 12-h light/dark cycle in a specific-pathogen free facility. There was no symptom of infection in dams and pups all through the experiment. Regular chow (formula number D06121301, Research Diets Inc., New Brunswick, NJ) was grinded into a fine powder and placed into a feeding basket (SN-950; Shimano Manufacturing, Co., Ltd., Tokyo, Japan). Dams were divided into two groups at 11.5 dpc. The NN group was fed powdered regular chow *ad libitum* (20 dams). Based on data obtained on the previous day, the daily energy intake of the UN group (20 dams) was restricted to 60% that of NN dams from 11.5 dpc to the day before the delivery of pups (17.5 dpc). In the morning of 18.5 dpc, 2.5 g of powdered standard diet was supplied to UN dams, followed by 3.5 g of extra food in the evening of 18.5 dpc just before the night of parturition to prevent the consumption of pups. NN and UN dams were both fed *ad libitum* after delivery, with powdered chow being changed to sticks 2.5 days after delivery.

The same dams nursed pups. In UN dams, the number of pups was adjusted to 4 per litter at 1.5 days of age (d) for the purpose of inducing RG during the lactation period (UN-RG pups; 60 randomly selected pups)(**Figure 1B**). In NN dams, the number of pups was adjusted to 8 per litter at 1.5 d as the control (NN pups; 60 randomly selected pups)(**Figure 1B**). Male pups were selected, and female pups were only included when matching odd numbers of 4 or 8 per litter. Rodent Lab Diet EQ 5L37 (Japan SLC, Inc., Hamamatsu, Japan) was supplied after weaning. A HFD containing 60% lipids (formula number D12492, Research Diets Inc.) was supplied to all pups from 9 to 16.5 wks. At 9 wks, we randomly selected 15 pups each from NN pups and UN-RG pups (a total of 30 pups). Between 11 and 16.5 wks, TUDCA (Merck Japan Ltd., Tokyo, Japan) (TU) or vehicle distilled water (Veh) was orally administered by gastric lavage at 0.5 g/kg body weight per day as previously described (17, 18), resulting in four groups, i.e. NN-Veh (n=9), NN-TU (n=6), UN-RG-Veh (n=9), and UN-RG-TU (n=6) groups (**Figure 1B**).

All experimental procedures were conducted in accordance with standards of humane animal care and approved by the Animal Research Committee, Hamamatsu University School of Medicine (H20-014).

### Tissue Sampling

At 16.5 wks of age, all pups in the independent experimental cohort were decapitated and samples of blood and epididymal adipose tissue were collected. Sampling was systematically performed from 9:00AM to 3:00PM under feeding by a trained technician blinded to the study. To measure adipose tissue weights, we used a GX-600 (A&D Company, Limited, Tokyo, Japan) with a minimum weighing value of 0.001g and standard deviation of 0.001 g. Some samples of adipose tissue were fixed in 10% formaldehyde (0.1 M sodium cacodylate buffer, pH 7.4) and embedded in paraffin for a morphological analysis. Tissue orientation was not taken into consideration when embedding the tissue. The remaining tissue was snap frozen using liquid nitrogen in blocks and stored at –80°C for mRNA extraction.

### Blood Sampling and Measurement

Immediately after the decapitation of pups, blood glucose levels were measured with ACC-CHEK Compact Plus (Roche Diagnostics Japan, Tokyo, Japan) and blood samples were collected into heparin-coated glass tubes and centrifuged at 1200×*g* at 4°C for 15 min. The plasma obtained was aliquoted and stored at –30°C until assayed. Total cholesterol, triglyceride, and HDL cholesterol levels were measured using FUJI DRI-CHEM 3500 (FUJIFILM Holdings Co., Tokyo, Japan).

### Microarray Analysis

Aliquots (250 ng) of total RNA obtained from 4 animals per group at 16.5 wks were individually converted to cDNA and labeled with Clariom™ S Assay, mouse (Thermo Fisher Scientific, Inc., cat.# 902930) and GeneChip™WT PLUS Reagent Kit (Thermo Fisher Scientific, Inc., cat.# 902281) according to the manufacturer’s instructions. Hybridization, washing, and staining were performed using the Hybridization, Wash, and Stain Kit (Thermo Fisher Scientific, Inc., cat.# 900720), GeneChip™ Hybridization Oven 645 (Thermo Fisher Scientific, Inc.), and GeneChip™ Fluidics Station 450 (Thermo Fisher Scientific, Inc.), according to the manufacturer’s protocols. After washing, Array Strips were analyzed using GeneChip™ Scanner 3000 7G (Thermo Fisher Scientific, Inc.). Data were validated using Expression Console™ and Transcriptome Analysis Console™ Software (Thermo Fisher Scientific, Inc.). A cut-off point of ≤-1.3 or ≥1.3 of the linear fold change and P-values was used. We submitted our microarray data, which was approved under the accession number GSE18830 to the GEO repository.

### Gene Enrichment, Heatmap, and Principal Component Analyses

A gene enrichment analysis of our list of genes from microarray data was performed to identify which molecular functions and biological processes were overrepresented (20). We used the Metascape online tool (https://metascape.org/gp/index.html#/main/step1) for the enrichment analysis of our gene list. Metascape provides automated meta-analysis tools to understand common and unique pathways within a group of orthogonal target-discovery studies. The terms of individual GO represent biological processes, cellular components, and molecular function categories, as well as Kyoto Encyclopedia of Genes and Genomes pathways, based on the Metascape online tools (21).

Heatmap and principal component analyses of the NN-Veh, UN-RG-Veh, and UN-RG-TU groups were performed using GraphPad Prism 9 (GraphPad Software, San Diego, CA, USA).

### Quantitative RT-PCR Analysis of Adipose Tissue

Total RNA was extracted from subcutaneous adipose tissue as previously described (13). Gene expression was evaluated by quantitative RT-PCR using the High Capacity RNA to cDNA Master Mix (catalog number 4390777; Applied Biosystems, Foster city, CA) and SYBR Green PCR Master Mix (catalog number 4309115; Applied Biosystems), according to the manufacturer’s recommendations. 18S ribosomal mRNA expression was used as an internal control. The primers used are listed in **Table 1**.

**Table 1:** Forward and revers primers used in quantitative RT-PCR.

	Genebank accession number	Forward	Reverse
*Saa3*	NM_011315.3	AACTATGATGCTGCCCGGAG	GCTCCATGTCCCGTGAACTT
*Ubd*	NM_023137.3	TCCGAGTTCGAAGATCCAGC	CTTCCAGCTTCTTTCCGTTGC
*S100a8*	NM_013650.2	ACTTCGAGGAGTTCCTTGCG	TGCTACTCCTTGTGGCTGTC
*Hpx*	NM_017371.2	TCCTGGGATCAGCCTTGAGA	ACCTCTGTCCATGTTGCCTG
*Casp1*	NM_009807.2	ACTGACTGGGACCCTCAAGT	GCAAGACGTGTACGAGTGGT
*Agt*	NM_007428.4	TTGGCGCTGAAGGATACACA	GATGTATACGCGGTCCCCAG
*Ptgs2*	NM_011198.4	TGAGTGGGGTGATGAGCAAC	AAGTGGTAACCGCTCAGGTG

### Histological Assessment of Adipose Tissue

Adipose tissue blocks embedded in paraffin were cut into 3-µm-thick sections. A macrophage-specific F4/80 rat monoclonal antibody (MCA497GA, AbD Serotec, Kidlington, UK) was applied to the sections. Detection was performed with a polymer detection kit (ChemMate EnVision™; Dako Japan, Tokyo, Japan) according to the manufacturer’s instructions, followed by a reaction with 3,3’-diaminobenzidine and counterstaining with hematoxylin.

Four microscopic fields (0.6 mm^2^ × 4) were arbitrarily selected in each section, and the number of positive cells was counted.

### Statistical Analysis

Data are expressed as means ± standard deviations. The significance of differences between two mean values was assessed using the Student’s *t*-test or the Mann-Whitney U test, where appropriate. GraphPad Prism Version 9 (GraphPad Software, San Diego, CA) was used for statistical calculations. The significance of differences among four mean values was assessed using ANOVA with Tukey’s multiple comparison test. A P-value of less than 0.05 was considered to be significant.

## Results

### Body Weight and Energy Intake Changes in Dams and Pups With or Without UN Followed by RG

**Figure 2:**
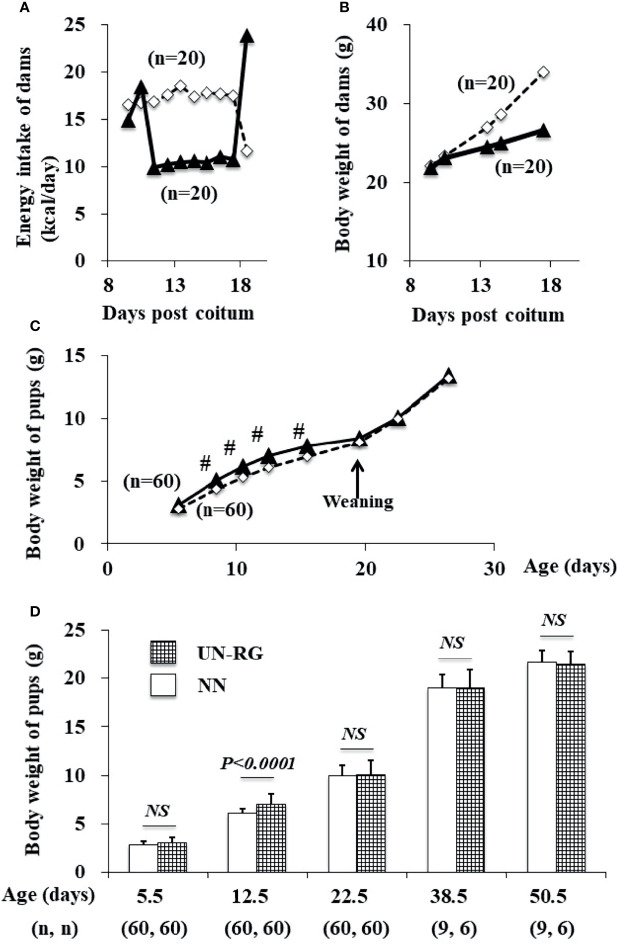
Changes in the energy intake **(A)** and body weight **(B)** of dams during pregnancy and in the body weight of pups **(C, D)** before HFD. White rectangles indicate NN dams **(A, B)** and pups **(C)**. Black triangles indicate UN **(A, B)** dams and UN-RG pups **(C)**. Means of the data were plotted **(A–C)**. ^#^P < 0.01. NS, no statistical significance.

Mean body weight from 8.5 to 16.5 d was significantly higher in UN-RG pups than in NN pups (**Figures 2C, D**). Similar growth patterns were observed in UN-RG and NN pups after weaning (**Figure 2C**).

**Figure 3:**
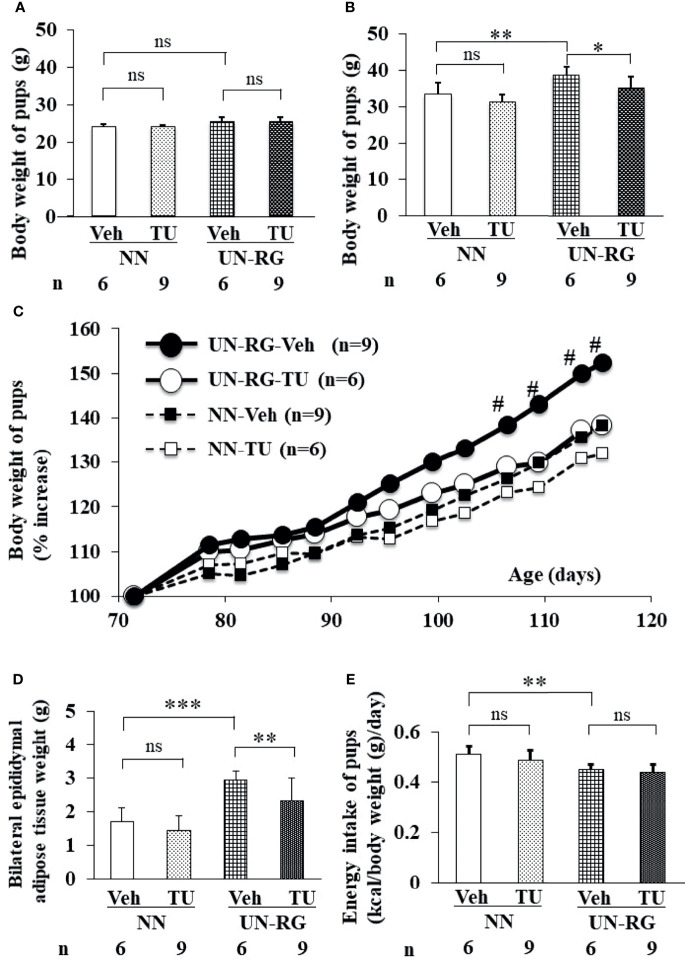
Changes in the body or tissue weight **(A–D)** and energy intake **(E)** of pups before **(A)** and after **(B–E)** HFD. Body weight [**(A)**; 9 wks] [**(B)**; 16.5 wks] [**(C)**; 9-16.5 wks],, epididymal adipose tissue weight [**(D)**; 16.5 wks), and energy intake [**(E)**; 15 wks]. Tissue sampling was performed at 16.5 wks. ^#^P < 0.01 *vs* NN-Veh. *P < 0.05; **P < 0.01; ***P < 0.001. NS, no statistical significance. Means of the data were plotted **(C)**.

After HFD, body weights were significantly higher in UN-RG-Veh pups than in NN-Veh pups (**Figures 3B, C**). Body weights were significantly lower in UN-RG-TU pups than in UN-RG-Veh pups (**Figures 3B, C**). Similar results were also observed for epididymal adipose tissue weight (UN-RG-TU *vs* UN-RG-Veh) (**Figure 3D**). TU did not alter energy intake per body weight (NN-TU *vs* NN-Veh and UN-RG-TU *vs* UN-RG-Veh) (**Figure 3E**).

Blood lipid profiles and glucose levels are summarized in **Table 2**.

**Table 2:** Body weight, adipose tissue weight, and lipid and glucose levels in blood at 16 wks.

	NN		UN-RG	
	Veh (n = 9)	TU (n = 6)	Veh (n = 9)	TU (n = 6)
Body weight (g)	33.5 ± 3.0	31.3 ± 2.3^#^	38.6 ± 2.4^#^	34.6 ± 2.6*
Left epididymal adipose tissue weight (g)	0.9 ± 0.2	0.7 ± 0.2	1.5 ± 0.1^#^	1.1 ± 0.3*
Serum total cholesterol (mg/dl)	195.8 ± 53.6	149.9 ± 25.9^#^	248.2 ± 23.4^#^	187.6 ± 50.1*
Triglycerides (mg/dl)	151.0 ± 40.7	127.9 ± 24.4	130.0 ± 17.4	147.6 ± 25.4
Serum HDL cholesterol (mg/dl)	172.5 ± 50.8	139.3 ± 23.5^#^	219.0 ± 14.2	159.3 ± 42.8*
Blood glucose (mg/dl)	221.0 ± 10.2	187.7 ± 10.7^#^	221.3 ± 45.9	183.2 ± 25.1*

*P < 0.05 vs UN-RG-Veh. ^#^P < 0.05 vs NN- Veh.

### Microarray, Gene Enrichment, Heatmap, and Principal Component Analyses of Epididymal Adipose Tissues at 16.5 Wks

**Figure 4:**
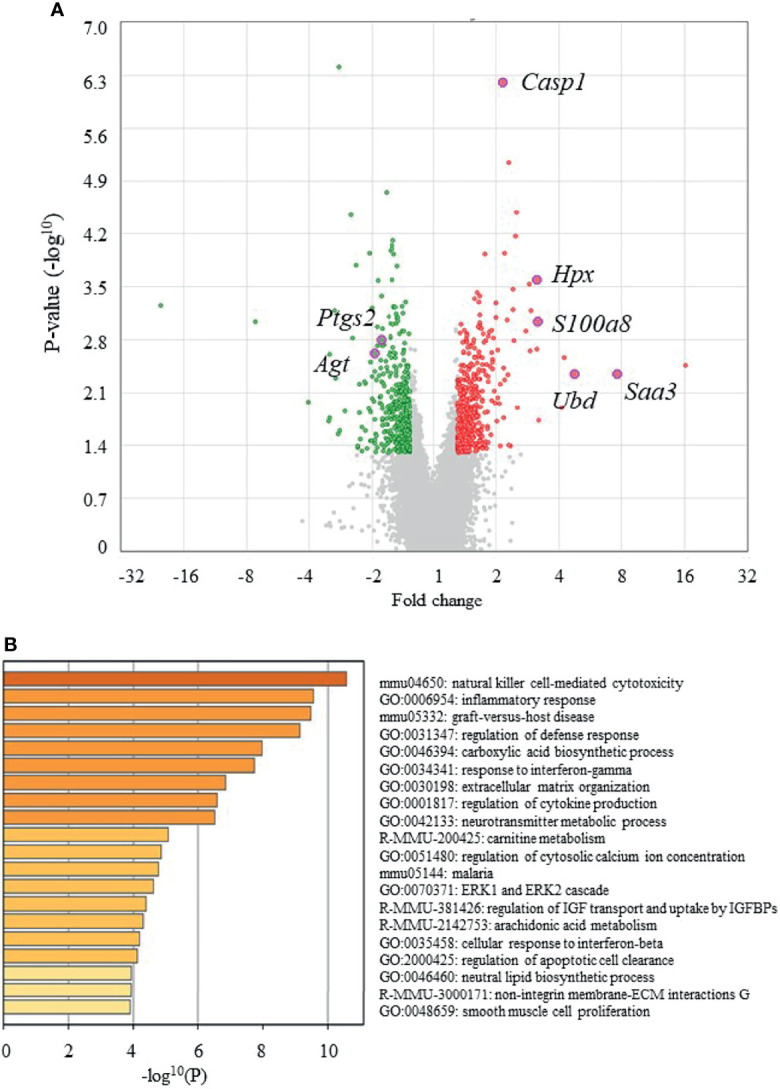
Volcano plots **(A)** and gene ontologies (GO) terms from a gene enrichment analysis **(B)** using microarray data from epididymal adipose tissues of UN-RG-Veh and NN-Veh pups (4 each) at 16.5 wks. The names of 7 GOI (**Table 3**) were plotted in volcano plots **(A)**.

**Figure 5:**
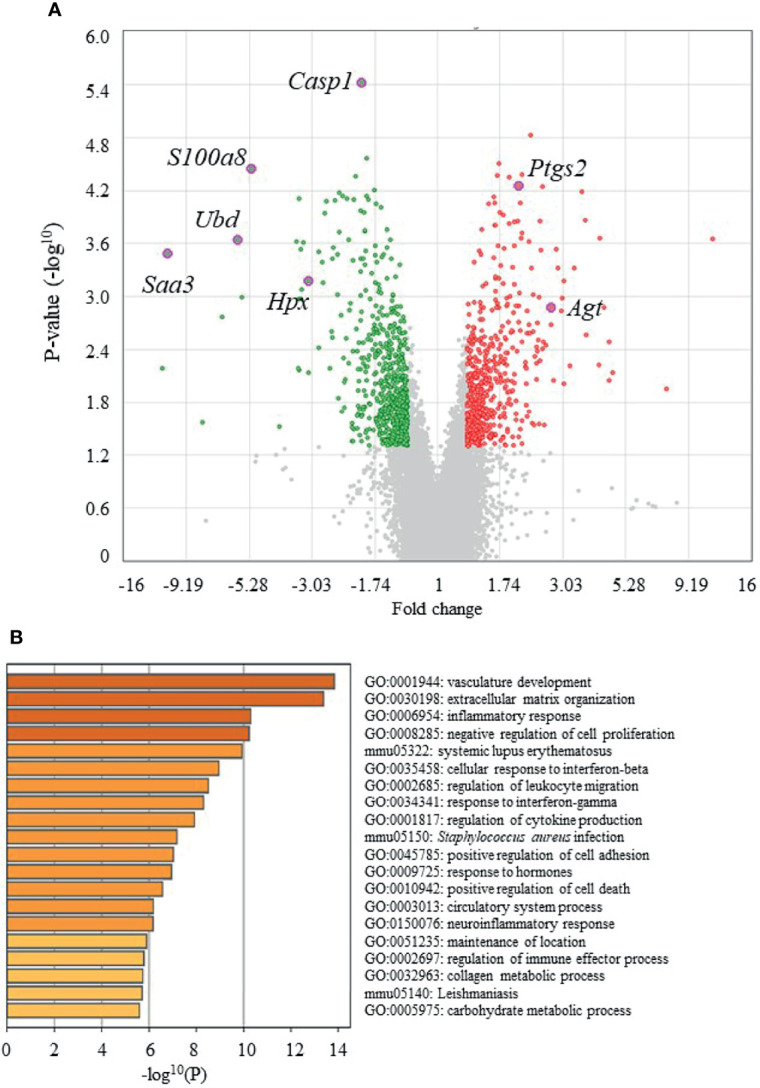
Volcano plots **(A)** and GO terms from a gene enrichment analysis **(B)** using microarray data from epididymal adipose tissues of UN-RG-TU and UN-RG-Veh pups (4 each) at 16.5 wks. The names of 7 GOI (**Table 3**) were plotted in volcano plots **(A)**.

**Figure 4A** shows volcano plots of microarray analyses of gene expression profiles in the epididymal adipose tissues of UN-RG-Veh and NN-Veh pups at 16.5 wks. The results of the gene enrichment analysis and resultant GO terms in ascending order of P-values are shown in **Figure 4B**. **Figure 5A** shows volcano plots of the microarray analysis of gene expression profiles in the epididymal adipose tissues of UN-RG-TU and UN-RG-Veh pups at 16.5 wks. The results of the gene enrichment analysis and resultant GO terms in ascending order of P-values are shown in **Figure 5B**. The representative genes which were not normalized by TU in UN-RG pups were summarized in **Supplementary Table 2**.

The following GO terms were commonly observed in the results of two gene enrichment analyses: 1) Inflammatory response, 2) Response to interferon-γ, 3) Regulation of cytokine production, and 4) Cellular response to interferon-β (**Figure 4B**, **5B**), suggesting their principal involvement in the changes observed in fat deposition in epididymal adipose tissue in response to UN-RG and/or TU. We have no clear explanation why these 4 GO terms commonly observed in two gene enrichment analysis and speculate that they may represent the pathways being involved in the plasticity of adipose tissue deposition in pups with UN-RG and TU. Representative changes in the gene expression profiles of the 4 GO terms are summarized in **Supplementary Table 1**.

**Figure 6:**
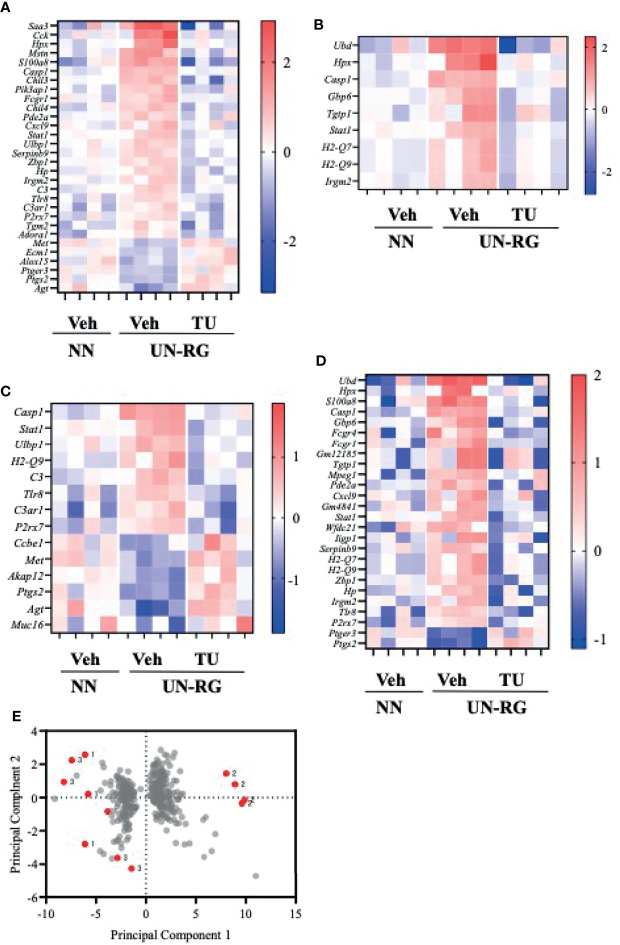
Heatmaps of the mRNA expression of representative genes from 4 GO terms **(A–D)** and a principal component analysis **(E)** of NN-Veh, UN-RG-Veh, and UN-RG-TU pups (mean of 4 pups each). **A**) Inflammatory response, **B**) Response to interferon-γ, **C**) Regulation of cytokine production, and **D**) Cellular response to interferon-β. These 4 GO terms were commonly observed in two gene enrichment analyses, i.e. **Figures 4B** and **5B**. **(E)** A principle component analysis: gray dots indicate principle scores, while red dots show the loading of NN-Veh (1), UN-RG-Veh (2), and UN-RG-TU (3). There was a tendency that NN-Veh (1) and UN-RG-TU (3) showed similar distribution in comparison with the distribution of UN-RG-Veh (2).

**Figure 6** shows heatmaps of relative fold increases in the expression of representative genes in the 4 newly identified GO terms in NN-Veh, UN-RG-Veh, and UN-RG-TU pups. The results obtained showed that the expression of genes up-regulated by UN-RG was suppressed by TU and vice versa. However, we were unable to obtain clear patterns for heatmaps by clustering related genes (data not shown); therefore, we simply listed gene names on the vertical axis (**Figures 6A–D**). The principal component analysis revealed similar changes in gene expression patterns between NN and TU in comparison with UN (**Figure 6E**).

**Figure 7:**
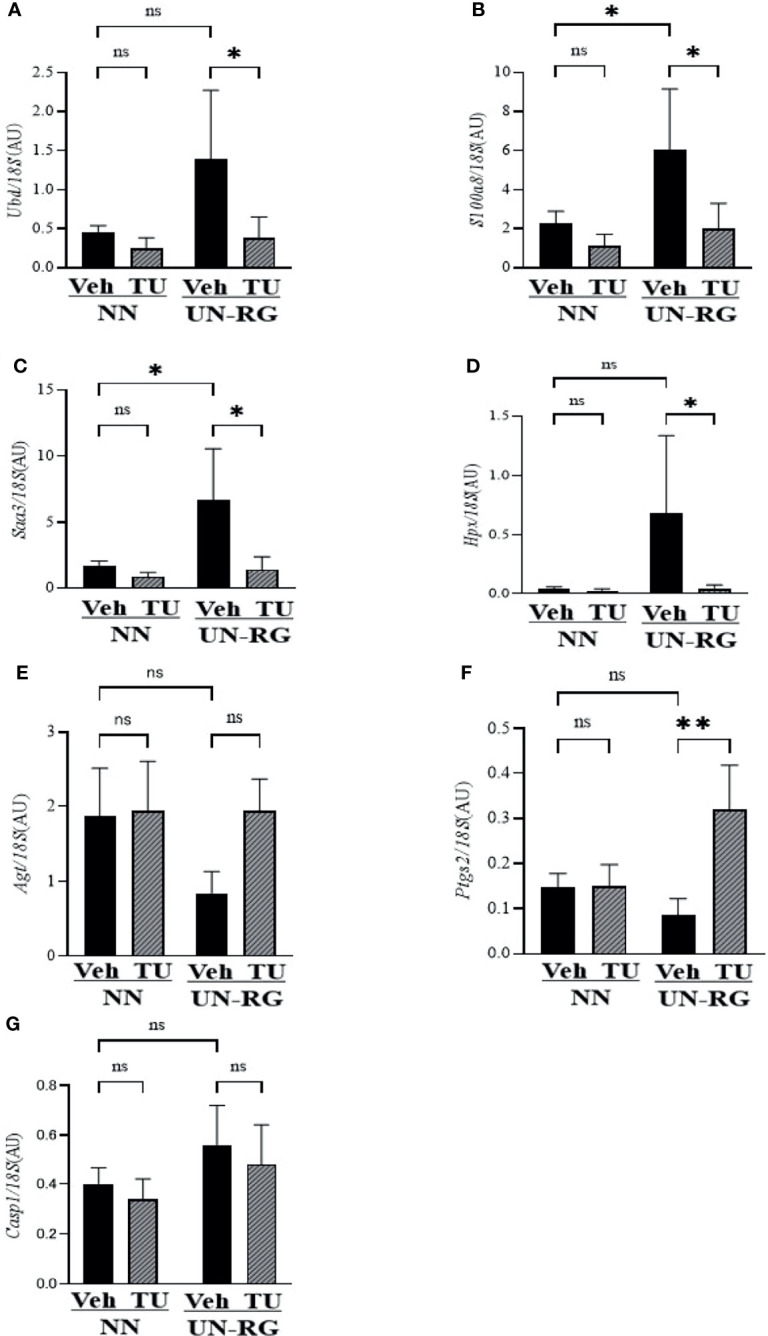
Quantitative RT-PCR analysis of the mRNA expression of seven GOI (**Table 3**) in epididymal adipose tissues of NN-Veh, NN-TU, UN-RG-Veh, and UN-RG-TN pups at 16.5 wks. *P < 0.05; **P < 0.01. ns, no statistically significant.

**Table 3** summarizes 7 genes of interest (GOI) that were observed at least twice in these 4 GO terms and also met the conditions of a ≤−2 or ≥2 linear fold change and P-value <0.05. The quantitative PCR analysis confirmed that UN-RG (*vs* NN-Veh) significantly up-regulated the gene expression of *Saa3*, *Ubd*, *S100a8*, and *Hpx*, significantly down-regulated the gene expression of *Agt*, and did not significantly affect the gene expression of *Casp1* and *Ptgs2* (**Figure 7**). In contrast, TU induced significantly down-regulated the gene expression of *Saa3*, *Ubd*, *S100a8*, and *Hpx*, significantly up-regulated the gene expression of *Agt* and *Ptgs2* and did not significantly affect the gene expression of *Casp1* (UN-RG-Veh *vs* UN-RG-TU) in UN-RG pups (**Figure 7**). In NN pups, TU did not significantly affect the expression of any of the 7 genes examined (NN-Veh *vs* NN-TU) (**Figure 7**).

**Table 3:** List of genes of interests (GOI).

Gene symbol	NN-Veh *vs* UN-RG-Veh		UN-RG-Veh *vs* UN-RG-TU	
	Fold change	P-value	Fold change	P-value
*Saa3*	7.60	0.0045	-10.88	0.0003
*Ubd*	4.73	0.0045	-5.86	0.0002
*S100a8*	3.17	0.0009	-5.19	<0.0001
*Hpx*	3.14	0.0003	-3.14	0.0007
*Casp1*	2.15	<0.0001	-1.96	<0.0001
*Agt*	-1.94	0.0024	2.73	0.0013
*Ptgs2*	-1.8	0.0016	2.05	<0.0001

These seven genes were observed at least twice in four different gene ontologies (GO), i.e. 1) Inflammatory response, 2) Response to interferon-γ, 3) Regulation of cytokine production, and 4) Cellular response to interferon-β, and also met the conditions of a ≤−2 or ≥2 linear fold change and P-value <0.05. These four GO were commonly observed in two GO enrichment analyses (UN-RG-Veh vs NN-Veh and UN-RG-TU vs UN-RG-Veh) using data from the microarray analysis of epididymal adipose tissue.

### Macrophage Infiltration in Epididymal Adipose Tissue

**Figure 8:**
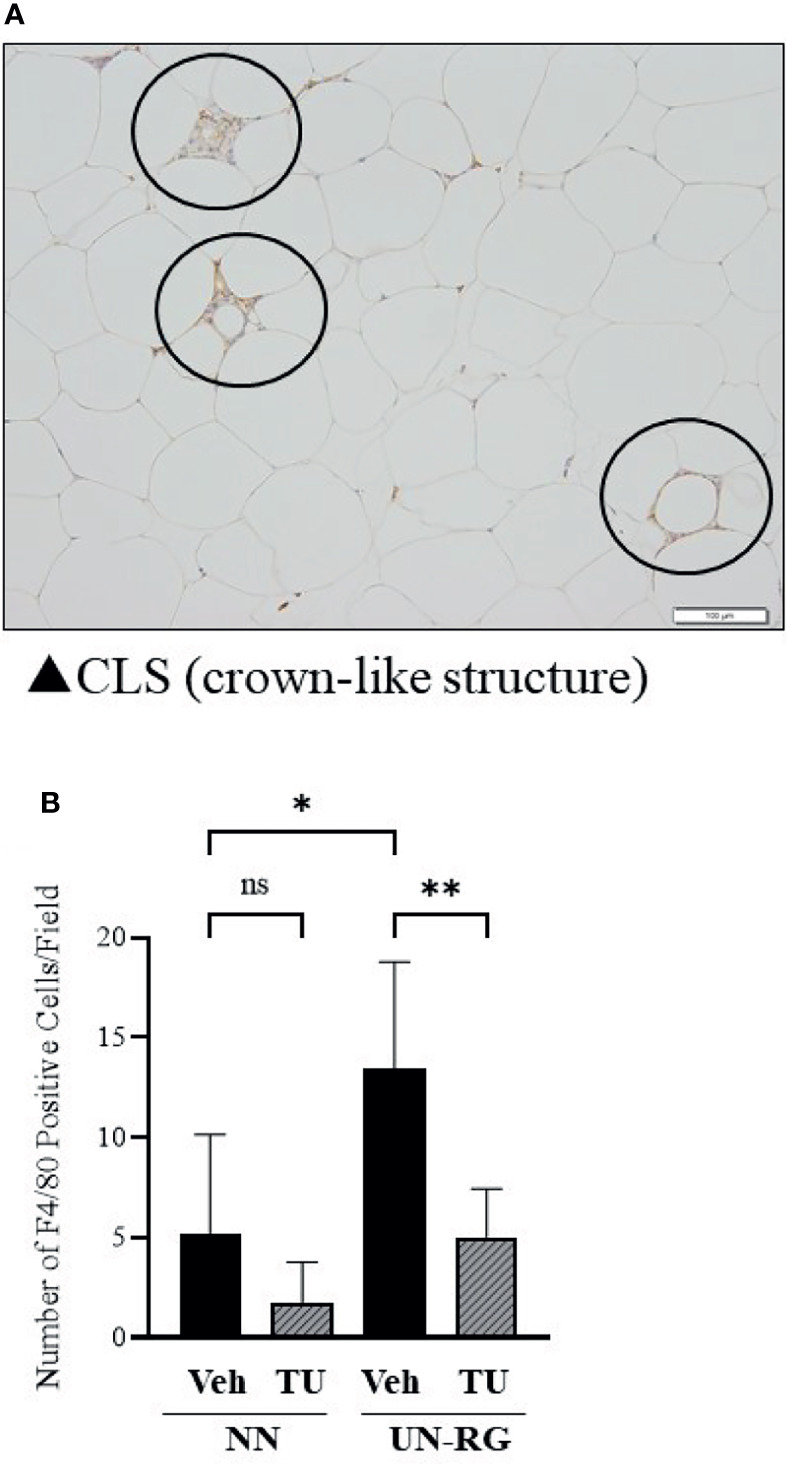
Immunohistochemical detection of macrophages in epididymal adipose tissue. Circles indicate immunostaining for F4/80 in crown-like structures of epididymal adipose tissue from UN-RG-Veh pups at 16.5 wks of age **(A)** and the average number of F4/80-positive cells in the epididymal adipose tissue of NN-Veh, NN-TU, UN-RG-Veh, and UN-RG-TU pups **(B)**. The white bar indicates 100 μm. *P < 0.05, **P < 0.01.

Crown-like structures were detected in the epididymal adipose tissue of pups fed the HFD at 16.5 wks of age (**Figure 8A**). Most of these crown-like structures were immunohistochemically positive for F4/80, corresponding to infiltrated or resident macrophages (**Figure 8A**). The mean number of F4/80-positive cells was significantly higher in UN-RG-Veh pups than in NN-Veh pups (**Figure 8B**). TU induced a significant decrease in the mean number of F4/80-positive cells in UN-RG pups (UN-RG-Veh *vs* UN-RG-TU) (**Figure 8B**).

## Discussion

Increasing evidence supports RG being causatively associated with obesity and/or metabolic syndrome in later life, particularly in neonates born small (6–10); however, the benefits of RG for later neurodevelopment supports its encouragement in infants born preterm (10). In the present study, we newly established a mouse model of RG during the lactation period following UN (UN-RG) (**Figures 1**, **2**), by modifying our previous model of UN (13–18). UN-RG pups developed diet-induced obesity (**Figure 3**) as well as metabolic disorders (**Table 2**), mimicking the phenotype of diet-induced obesity and associated metabolic syndrome causatively connected with rapid infantile growth (6–10).

As shown **Figures 2C, D**, mean body weight from 8.5 d to 16.5 d was significantly higher in UN-RG pups than in NN pups, but similar at weaning 18.5 d. Our previous study showed that mean body weight of simple UN pups from fetal period (18.5 dpc) to early infantile period (3.5 d) was significantly lower than in NN pups (13). Therefore, it is noted that the present UN-RG pups experienced drastic changes from relatively small fetal body weight to large neonatal body weight (5.5 d-16.5 d), which we believe is an appropriate animal model of the small human babes who experienced rapid growth after birth and suffered from NCDs in later life, as reported by epidemiological studies (6, 7), corresponding to ‘Mismatch’ hypothesis in DOHaD theory (11, 12).

In the present study, we focused to design the animal model for mimicking the epidemiological outcomes of infantile rapid growth of small neonate (6, 7) and did not simultaneously carry out only RG pups experiment due mainly to animal facility limitation. However, we developed UN-RG animal model, considering that risk of NCDs resulted from RG in small human newborns applies well to ‘Mismatch hypothesis’ (11, 12), one of central hypotheses of DOHaD theory. ‘Mismatch hypothesis’ is based on the concept that small fetuses developmentally adapted to the low energy supply and caused ‘Mismatch’ to the abundant energy supply after birth, resulted in predisposition to obesity and associated metabolic disorders (11, 12). It is undeniable that the adult phenotype of UN-RG pups might be similar to that of RG pups. It is also unclear if an experience of the UN affected the degree of RG, although it was speculated that main contributor of RG was increased breast feeding in UN-RG pups. Nevertheless, we would like to note that simple RG pups model is a clearly different research target from UN-RG pups, because simple RG pups mimic babies with normal birth weight who experienced rapid infantile growth, which is not directly associated with ‘Mismatch hypothesis’ (11, 12).

We performed a comprehensive analysis of gene expression profiles in epididymal adipose tissue as a visceral fat pad. We initially investigated the effects of UN-RG on gene expression profiles in epididymal adipose tissue using a microarray analysis (NN-Veh *vs* UN-RG-Veh), displayed the results in volcano plots (**Figure 4A**), and then conducted a gene enrichment analysis, which identified the top 20 GO terms in ascending order of P-values (**Figure 4B**). Since TU significantly attenuated diet-induced obesity (**Figures 3B, C**) and epididymal fat deposition (**Figure 3D**), we examined its effects on gene expression profiles in epididymal adipose tissue using a microarray analysis (UN-RD-Veh *vs* UN-RG-TU). The results obtained were displayed in volcano plots (**Figure 5A**). A subsequent gene enrichment analysis identified the top 20 GO terms in ascending order of P-values (**Figure 5B**).

In 4 inflammatory GO terms, heatmap analysis of the mRNA expression of representative genes showed the genes of increased expression by UN showed a decrease by TU; while the genes of decreased expression by UN showed an increase by TU (**Figures 6A–D**), which suggests their coordinated contribution to the inflammatory changes observed in fat pads in response to UN and TU. However, we were unable to obtain a clear pattern for heatmaps by clustering related genes and, thus, simply listed gene names on the vertical axis (**Figures 6A–D**). We then performed a principal component analysis, which revealed similar changes in gene expression patterns between NN and TU in comparison with UN (**Figure 6E**).

We subsequently acknowledged the following 4 GO terms: 1) Inflammatory response, 2) Response to interferon-γ, 3) Regulation of cytokine production, and 4) Cellular response to interferon-β, all of which were commonly detected in the two different GO terms groups (**Figures 4B**, **5B**), for the purpose of identifying potential critical pathways contributing to the plasticity observed in the regulation of fat deposition, which responded not only to UN, but also to TU (**Figures 3B–D**). All 4 GO terms were directly related to inflammatory reactions, suggesting that inflammation is a key phenomenon in the plasticity of the regulation of fat deposition in this animal model. Top GO term of natural killer cell-mediated cytotoxicity (UN-RG-Veh *vs* NN-Veh) also supports a possible involvement of inflammation (**Figure 4B**) (22, 23). It is noted that GO terms of carnitine metabolism and regulation of apoptotic cell clearance, direct pathways of lipid metabolism, were demonstrated by comparison between NN-Veh and UN-RG-Veh (**Figure 4B**), but not between UN-RG-Veh and UN-RG-TU (**Figure 5B**). We speculate that UN-RG might induce lipid metabolism as well as inflammation and that TU might affect mainly inflammatory regulation. More investigation is necessary to prove this speculation. TUDCA is an alleviator of ER stress, and it is noted that changes of *Kdelr 3*, ER protein retention receptor 3, gene expression was observed by comparison between NN-Veh and UN-RG-Veh (original microarray data is available by referring GSE18830 of the GEO repository).

Recent studies reported that obesity and its metabolic disruption are closely associated with low-level systemic inflammation, based upon which the concept of ‘metaflammation’ was proposed (24–27). Hotamisligil et al. (2017) proposed adipose tissue, the liver, pancreas, and brain as representative organs of ‘metaflammation’ (27). Lauterbach et al. (2017) suggested the principal involvement of tissue-resident macrophages in ‘metaflammation’ (28). Therefore, we performed an immunohistochemical analysis of macrophages in epididymal adipose tissue (**Figure 8A**) and found that UN-RG induced significant increases in the numbers of macrophages, while TU reversed this effect (**Figure 8B**, **Supplementary Figure 2**). These results support the critical contribution of ‘metaflammation’ to the adjustment of fat deposition in this animal model. There was a tendency of increase in the gene expression ratio of CD11c/CD163 in epididymal adipose tissue of UN-RG pups at 16.5 wks, but statistically not significant (**Supplementary Figure 1**). It is a future aim of the study to investigate the characteristics of these macrophages.

Previous studies proposed the potentially long-lasting embryonal memory of immune cells, including tissue-resident macrophages. Mass et al. (2021) suggested that fetal-derived immune cells are the prime transmitters of the long-term consequences of prenatal adversities, causing inflammatory, degenerative, and metabolic disorders (29). The potential commitment of erythro-myeloid progenitors produced in the extra-embryonic yolk sac to the ability to establish long-lasting immunological memory has been proposed (29–31); however, the exact mechanisms by which the memory of tissue resident macrophages is transferred to older generation macrophages remain unclear. Troger et al. (2013) demonstrated that the ability of the innate immune system of the growth-restricted fetus to mount an immune response was weakened after birth (32). Gotz et al. (2007) reported that prenatal stress may alter the number and proliferation of lymphocytes as well as the cytotoxicity of natural killer cells in the blood of male adult rat offspring (33). The gene expression profiles obtained in the present study also support the causative relationship of an environmental imbalance during the early critical period with the predisposition of adults to metaflammation; however, we did not detect immune memory in the macrophages of adipose tissue in this animal model.

In the search for the main molecular mechanisms in this animal model, we identified 7 GOI: *Saa3*, *Ubd*, *S100a8*, *Hpx*, *Casp1*, *Agt*, and *Ptgs2*, which were observed at least twice in the 4 GO terms that we selected in the present study, and also meet the conditions of a ≤−2 or ≥2 linear fold change and P-value <0.05 (**Table 3**). Quantitative RT-PCR reconfirmed the changes in their gene expression profiles in response to UN-RG and TU, except for *Casp1* (**Figure 7**). We currently have no clear explanation for the discrepancy regarding *Casp1*. Although limited information is currently available on their contribution to metaflammation in the DOHaD scheme, these genes play the following critical roles in the regulation of general inflammation. *Saa3* (34), *Agt* (35), and *Ptgs2* (36) are all involved in the inflammation of obesity. *Ubd* has been implicated in inflammation in nonalcoholic fatty liver disease (37). *Hpx* functions as an antioxidant and regulates inflammation (38). *Casp1* is an effector of NLRC4 inflammasomes (39) and converts pro-IL-1β and pro-IL-18 into their biologically active forms (40). Regarding the activation of macrophages, *Saa3* has been shown to promote the infiltration of macrophages in adipose tissue (41), while *S100a8* plays a central role in obesity-promoting macrophage-based inflammation (42) and induces the secretion of cytokines from mononuclear cells (43). We would like to speculate possible a series of reactions by these genes in macrophage-based metaflammation, 1) induction of macrophage migration by *Saa3*, 2) induction of the secretion of cytokines from macrophage by *S100a8*, 3) activation of cytokines secreted from macrophage by *Casp1*, and 4) possible connection to insulin sensitivity by *Ubd* nd/or *Hpx*. More intensive studies are needed to clarify the direct involvement of these genes in the developmentally-induced augmentation of macrophage infiltration and metabolic disruption in the adipose tissue of this animal model. Epigenetic changes in these seven GOI are now under investigation by our research group. It is our future aim to investigate the involvement of these GOI in the developmentally-induced regulation of fat deposition and macrophage infiltration in this animal model. Our previous study showed that gene expression of *MCP-1* and *TNF-α* correlated with subcutaneous adipose tissue weight at 17 wks of simple UN pups, but not in NN pups (16), indirectly suggested a possible association between fat deposit and expression of inflammatory cytokines. We have no clear explanation why these two genes were not identified as GOI in UN-RG pups, because we did not carry out microarray analysis at that time and *MCP-1* and *TNF-α* were selected as representative inflammatory cytokines in UN pups, and also because the present UN-RG pups was designed to represent ‘Mismatch hypothesis’ in DOHaD theory (11, 12), quite different from simple UN pups at that time.

There are some limitations in the present study. We investigated 4 groups of pups, i.e. the NN-Veh, NN-TU, UN-RG-Veh, and UN-RG-TU groups, but not a NN-RG-Veh or NN-RG-TU group because of the restrictions of our animal facility accommodation. It is noted that Lizarraga-Mollinedo reported that different growth speed in the pups with undernourishment *in utero* showed conflicting levels of gene expression patterns in adipose tissue compared to normally nourished pups by using rat animal model (44). We did not investigate a condition without HFD due to the same animal facility limitation. We did not investigate time course of gene expression profiles. Furthermore, we were unable to obtain clear patterns for heatmaps by clustering related genes and, thus, simply listed gene names on the vertical axis of heatmaps (**Figures 6A-D**). We identified 7 GOI from 4 GO terms based on the results of microarray and gene enrichment analyses; however, we did not demonstrate their direct contribution to the programing of metaflammation in this animal model. We did not measure the plasma levels of inflammatory cytokines due to lack of the serum. Further study is needed to prove the involvement of 7 GOI in the pathophysiology of metaflammation. As for quantitative RT-PCR, 18S ribosomal mRNA expression was used as an internal control; however, we did not assess the effect of RG or TU on its expression. The main contributor of RG was supposed to be breastfeeding in this animal model; however, we could not assess the changes in breastfeeding of dams due to technical limitation. In the preset study, we focused on the assessment of epididymal adipose tissue, as a visceral fat, and did not investigate subcutaneous and retroperitoneal adipose tissues. It is a future aim of the study to investigate the changes in gene expression in subcutaneous and retroperitoneal adipose tissues.

In summary, we newly established a mouse animal model of RG during the lactation period following UN (**Figures 1**, **2**, **Table 2**), the offspring of which developed diet-induced obesity and metabolic disorders, which were significantly improved by TU (**Figure 3**), although we did not assess UN pups without GR. The microarray analysis and subsequent gene enrichment analysis (**Figure 4**, **Figure 5**) identified 4 GO terms of inflammatory pathways, indicating the critical contribution of chronic inflammation to developmentally-programed fat deposition and its amelioration by TU. The results of the heatmap and principal component analyses of the representative genes of the 4 GO terms (**Figure 6**), changes in the gene expression profiles of 7 GOI (**Table 3**, **Figure 7**) selected from 4 GO terms, and immunohistochemistry on macrophages (**Figure 8**) collectively support the proposal of the ‘Developmental Origins of Metaflammation’. Future epigenetic and immunological investigations, with a focus on 7 GOI, are needed to further establish this concept.

## Data Availability Statement

The datasets presented in this study can be found in online repositories. The names of the repository/repositories and accession number(s) can be found in the article/**Supplementary Material**.

## Ethics Statement

The animal study was reviewed and approved by the Animal Research Committee, Hamamatsu University School of Medicine (H20-014). Written informed consent was obtained from the owners for the participation of their animals in this study.

## Author Contributions

MS and YK-K performed and analyzed all experiments. MS and HI wrote the manuscript. MU, KM, and HI designed the study. NF-I, TO, and MasM performed histological examinations. MS, TO, KK, TI, and MadM supported data collection and animal experiments, sample preparation, and data analysis. NT, TU, MN, and YK-K supported data collection, discussions, and statistical analyses. MS, TO, KK, and TI supported data analyses and preparation of the manuscript. KM supported the gene enrichment analysis. All authors contributed to the article and approved the submitted version.

## Funding

This work was supported by JSPS KAKENHI Grant Numbers, JP20H03823, JP20K09666, and JP20K16886, and AMED under Grant Number JP20gm1310009.

## Conflict of Interest

The authors declare that the research was conducted in the absence of any commercial or financial relationships that could be construed as a potential conflict of interest.

## Publisher’s Note

All claims expressed in this article are solely those of the authors and do not necessarily represent those of their affiliated organizations, or those of the publisher, the editors and the reviewers. Any product that may be evaluated in this article, or claim that may be made by its manufacturer, is not guaranteed or endorsed by the publisher.
