# Supplementary material for: Comparative Analysis of Gene Expression Profiles in the Adipose Tissue of Obese Adult Mice With Rapid Infantile Growth After Undernourishment In Utero
Source: Front Endocrinol (Lausanne). 2022 Feb 24;13:818064. doi: 10.3389/fendo.2022.818064 (PMC8920555; doi:10.3389/fendo.2022.818064)
Supplement: Supplementary file 1 [file DataSheet_1.doc]

**Supplemental Table 1**. List of genes. Microarray analysis of epididymal adipose tissue (≤−2 or ≥2 linear fold change and P-value <0.05).

(A)

| GO:0035458 Cellular response to interferon-β | | | | |
| --- | --- | --- | --- | --- |
|  | NN-Veh  vs  UN-RG-Veh | | UN-RG-Veh  vs  UN-RG-TU | |
| Gene symbol | Fold change | P-value | Fold change | P-value |
| *Ubd* | 4.73 | 0.0045 | -5.87 | 0.0002 |
| *S100a8* | 3.17 | 0.0009 | -5.19 | 0.0000 |
| *Hpx* | 3.14 | 0.0003 | -3.14 | 0.0007 |
| *Casp1* | 2.15 | 0.0000 | -1.96 | 0.0000 |
| *Gbp10; Gbp6* | 1.78 | 0.0016 | -2.31 | 0.0001 |
| *Fcgr4* | 1.92 | 0.0038 | -2.01 | 0.0033 |
| *Ptgs2* | -1.8 | 0.0016 | 2.05 | 0.0001 |

(B)

| GO:0006954 Inflammatory response | | | | |  |
| --- | --- | --- | --- | --- | --- |
|  | NN-Veh  vs  UN-RG-Veh | | UN-RG-Veh  vs  UN-RG-TU | | |
| Gene symbol | Fold change | P-value | Fold change | P-value |  |
| *Saa3* | 7.6 | 0.0045 | -10.88 | 0.0003 |  |
| *Cck* | 4.22 | 0.0027 | -5.62 | 0.0010 |  |
| *S100a8* | 3.17 | 0.0009 | -5.19 | 0.0000 |  |
| *Mstn* | 2.93 | 0.0007 | -3.49 | 0.0002 |  |
| *Hpx* | 3.14 | 0.0003 | -3.14 | 0.0007 |  |
| *Chil3* | 1.78 | 0.0362 | -3.28 | 0.0002 |  |
| *Agt* | -1.94 | 0.0024 | 2.73 | 0.0013 |  |
| *Chil4* | 1.9 | 0.0102 | -2.36 | 0.0016 |  |
| *Casp1* | 2.15 | 0.0000 | -1.96 | 0.0000 |  |
| *Pik3ap1* | 1.9 | 0.0024 | -2.17 | 0.0014 |  |
| *Met* | -1.85 | 0.0047 | 2.08 | 0.0025 |  |
| *Ptgs2* | -1.8 | 0.0016 | 2.05 | 0.0001 |  |

(C)

| GO:0001817 Regulation of cytokine production | | | | |
| --- | --- | --- | --- | --- |
|  | NN-Veh  vs  UN-RG-Veh | | UN-RG-Veh  vs  UN-RG-TU | |
| Gene symbol | Fold change | P-value | Fold change | P-value |
| *Muc16* | -2.23 | 0.0472 | 3.71 | 0.0027 |
| *Agt* | -1.94 | 0.0024 | 2.73 | 0.0013 |
| *Casp1* | 2.15 | 0.0000 | -1.96 | 0.0000 |
| *Met* | -1.85 | 0.0047 | 2.08 | 0.0025 |
| *Ptgs2* | -1.8 | 0.0016 | 2.05 | 0.0001 |

(D)

| GO:0034341 Response to interferon-γ | | | | |
| --- | --- | --- | --- | --- |
|  | NN-Veh  vs  UN-RG-Veh | | UN-RG-Veh  vs  UN-RG-TU | |
| Gene symbol | Fold change | P-value | Fold change | P-value |
| *Ubd* | 4.73 | 0.0045 | -5.87 | 0.0002 |
| *Hpx* | 3.14 | 0.0003 | -3.14 | 0.0007 |
| *Casp1* | 2.15 | 0.0000 | -1.96 | 0.0000 |
| *Gbp10; Gbp6* | 1.78 | 0.0016 | -2.31 | 0.0001 |
| *Ptgs2* | -1.8 | 0.0016 | 2.05 | 0.0001 |

**Supplemental Table 2.** List of representative genes, the changes of which were not normalized by TU in UN-RG pups (UN-GR-Veh vs UN-GR-TU).

|  | NN-TU vs UN-RG-TU | |
| --- | --- | --- |
| Gene symbol | Fold change | P-value |
| *Actg2* | -2.62 | 0.0353 |
| *Cep55* | -2.16 | 0.0064 |
| *5730522E02Rik* | -2.04 | 0.001 |
| *Vsig4* | -2 | 0.0242 |
| *Gm10720* | 2.3 | 0.0466 |
| *Gm10800* | 3.01 | 0.0429 |

Microarray analysis of epididymal adipose tissue (≤−2 or ≥2 linear fold change and P-value <0.05).
