# Supplementary material for: Comparative Analysis of Gene Expression Profiles in the Adipose Tissue of Obese Adult Mice With Rapid Infantile Growth After Undernourishment In Utero
Source: Front Endocrinol (Lausanne). 2022 Feb 24;13:818064. doi: 10.3389/fendo.2022.818064 (PMC8920555; doi:10.3389/fendo.2022.818064)
Supplement: Supplementary file 2 [file Presentation_1.pptx]

## Slide 1
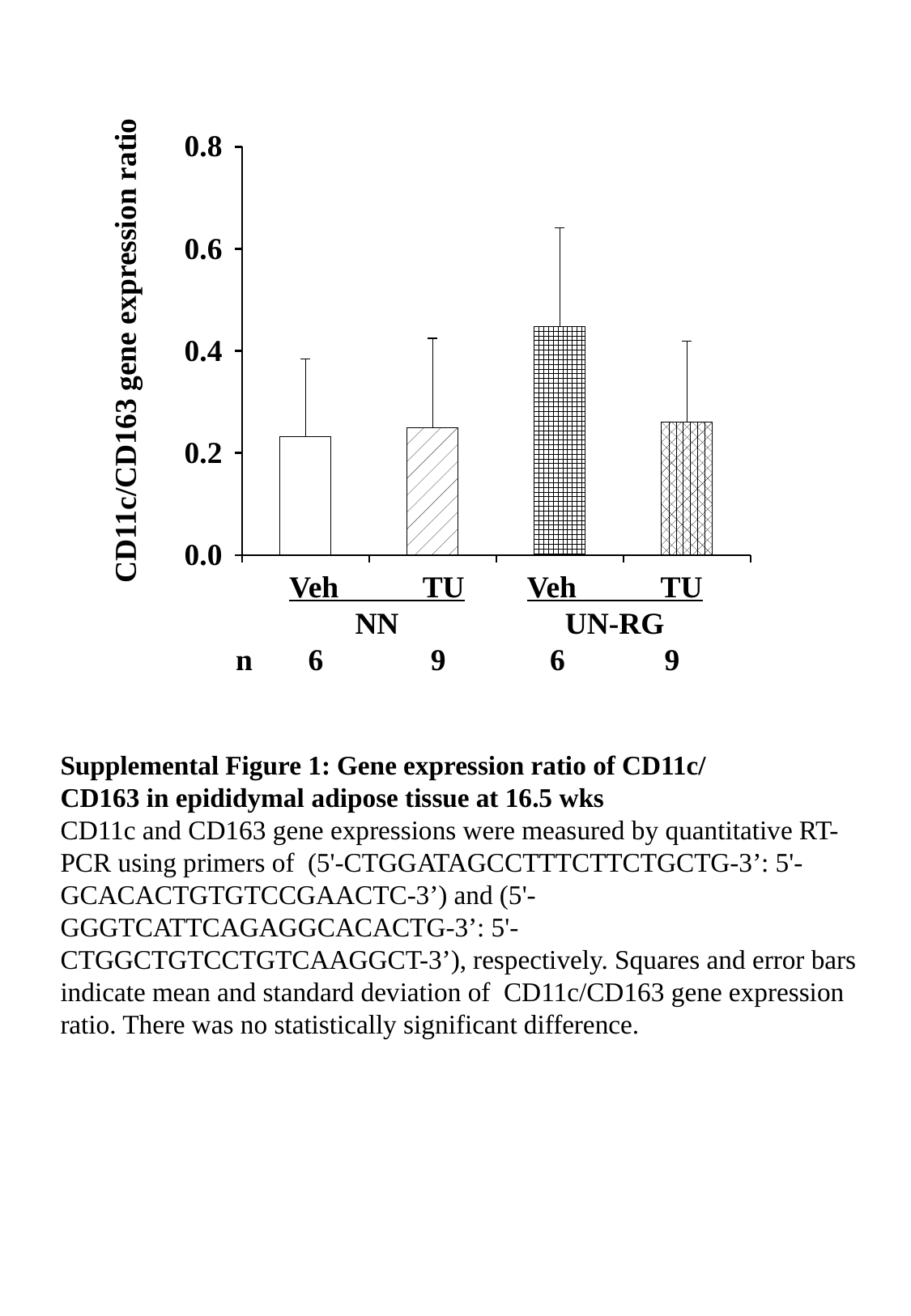

### Chart
| Category | 平均値 |
|---|---|
| C-Veh | 0.23258422433139325 |
| C-TUD | 0.24949730965133218 |
| CR-Veh | 0.4482666223677887 |
| CR-TUD | 0.26065752389209174 |CD11c/CD163 gene expression ratio
Veh TU
NN
6 　 9
Veh TU
UN-RG
6 　 9
n
Supplemental Figure 1: Gene expression ratio of CD11c/
CD163 in epididymal adipose tissue at 16.5 wks
CD11c and CD163 gene expressions were measured by quantitative RT-PCR using primers of (5'-CTGGATAGCCTTTCTTCTGCTG-3’: 5'-GCACACTGTGTCCGAACTC-3’) and (5'-GGGTCATTCAGAGGCACACTG-3’: 5'-CTGGCTGTCCTGTCAAGGCT-3’), respectively. Squares and error bars indicate mean and standard deviation of CD11c/CD163 gene expression ratio. There was no statistically significant difference.

## Slide 2
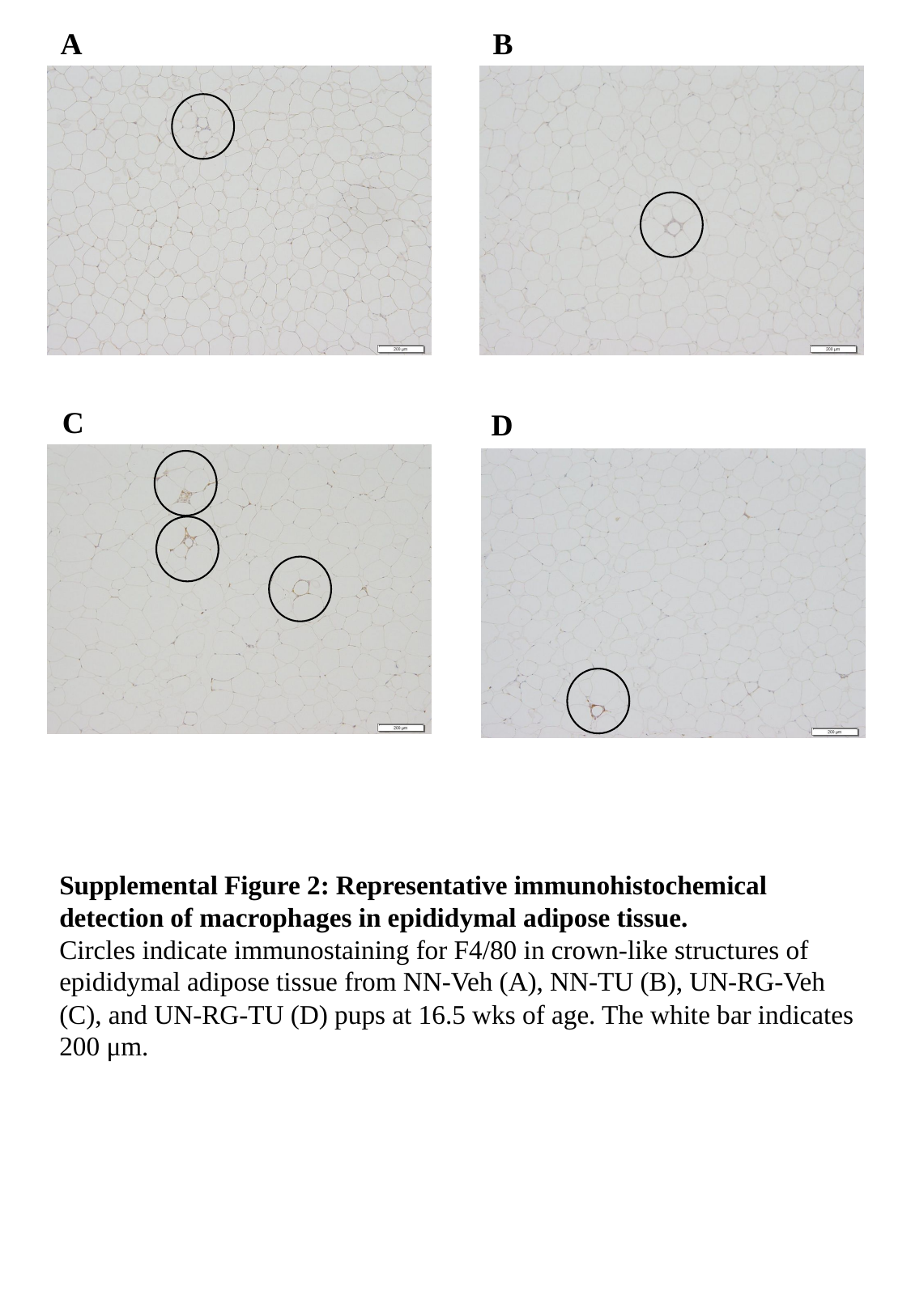

A
B
C
D
Supplemental Figure 2: Representative immunohistochemical detection of macrophages in epididymal adipose tissue.
Circles indicate immunostaining for F4/80 in crown-like structures of epididymal adipose tissue from NN-Veh (A), NN-TU (B), UN-RG-Veh (C), and UN-RG-TU (D) pups at 16.5 wks of age. The white bar indicates 200 μm.
